# Mini-review: The Role of the Cerebellum in Visuomotor Adaptation

**DOI:** 10.1007/s12311-021-01281-4

**Published:** 2021-06-02

**Authors:** Elinor Tzvi, Sebastian Loens, Opher Donchin

**Affiliations:** 1 Department of Neurology, University of Leipzig, Liebigstraße 20, 04103 Leipzig, Germany; 2 Institute of Systems Motor Science, University of Lübeck, Ratzeburger Allee 160, 23538 Lübeck, Germany; 3 Motor Learning Lab, Ben Gurion University of the Negev, Be’er Sheva, Israel

## Abstract

The incredible capability of the brain to quickly alter performance in response to ever-changing environment is rooted in the process of adaptation. The core aspect of adaptation is to fit an existing motor program to altered conditions. Adaptation to a visuomotor rotation or an external force has been well established as tools to study the mechanisms underlying sensorimotor adaptation. In this mini-review, we summarize recent findings from the field of visuomotor adaptation. We focus on the idea that the cerebellum plays a central role in the process of visuomotor adaptation and that interactions with cortical structures, in particular, the premotor cortex and the parietal cortex, may be crucial for this process. To this end, we cover a range of methodologies used in the literature that link cerebellar functions and visuomotor adaptation; behavioral studies in cerebellar lesion patients, neuroimaging and non-invasive stimulation approaches. The mini-review is organized as follows: first, we provide evidence that sensory prediction errors (SPE) in visuomotor adaptation rely on the cerebellum based on behavioral studies in cerebellar patients. Second, we summarize structural and functional imaging studies that provide insight into spatial localization as well as visuomotor adaptation dynamics in the cerebellum. Third, we discuss premotor — cerebellar interactions and how these may underlie visuomotor adaptation. And finally, we provide evidence from transcranial direct current and magnetic stimulation studies that link cerebellar activity, beyond correlational relationships, to visuomotor adaptation .

## Sensorimotor Learning vs. Sensorimotor Adaptation

To be able to master a new skill, you need to practice, sometimes for days. Improved performance of sensory-guided motor behavior is referred to as motor learning. This type of learning may take different forms, depending on the skill to be learned. For example, learning to play the piano requires integration of visual, auditory and fine-motor skills [1], whereas learning to play golf demands visuomotor coordination and error-based learning [2]. In the lab, researchers have designed experimental settings that differentially evoke different forms of motor learning. For instance, to study the acquisition of a new motor skill, a widely used task is the motor sequence learning task, which commonly relies on finger-tapping: a sequence of button presses learnt implicitly using for instance the serial reaction time task [3] or explicitly [4]. To study error-based learning, researchers have used paradigms that are based on sensorimotor adaptation. Adaptation in this context refers to the process of fitting an already learned motor program to new environmental circumstances [5], for example when trying to walk on a moving train. A meta-analysis of motor learning imaging studies showed that premotor cortex, supplementary motor area, superior parietal lobule and thalamus are more associated with sequence learning tasks, while the cerebellum and basal ganglia are more associated with sensorimotor adaptation tasks [6], suggesting that neural substrates of motor learning variants may differ.

## Visuomotor Adaptation Tasks

**Fig. 1:**
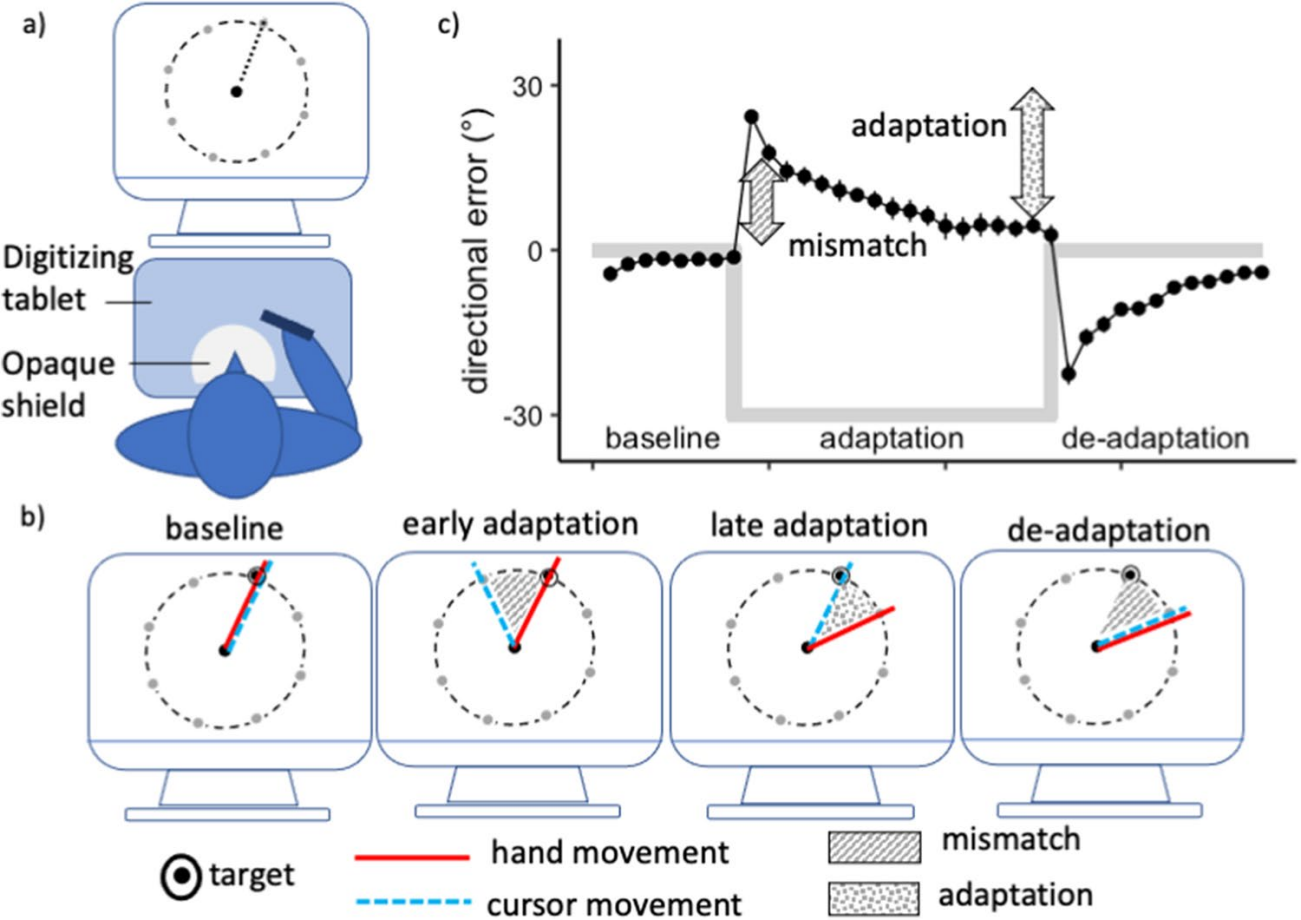
Visuomotor adaptation paradigm. (**a**) Experimental setup. Subjects perform the visuomotor adaptation task on a digitizing tablet. The view of their upper limbs is blocked by an opaque shield. Subjects observe their movements on a PC monitor. (**b**) The movement of the cursor on screen (dashed blue line) relative to the movement of the hand on the tablet (red line, not shown to the subjects) during the different stages of the experiment. (**c**) Directional error, hence the difference between the target position and the actual movement trajectory, over the course of the experiment. Thick gray bars indicate the changes in the applied perturbation

In a typical visuomotor adaptation task, participants perform simple reaching movements which are disturbed by an external manipulation. For example, in prism adaptation [7], subjects are instructed to point to a visual target while a visual displacement is introduced by a prism, leading to spatial errors. In a force-field adaption task [8], subjects perform movements with a robotic device which is subjected to perturbing forces, leading subjects to adapt their movements in order to hit the target. In a visuomotor rotation task, subjects perform center-out movements, commonly on a digitizing tablet (Fig. 1A). When the cursor movement, displayed on a computer screen, is shifted by an angular displacement, a mismatch between vision and proprioception leads subjects to adjust their movements (Fig. 1B). The initial disturbance in these tasks skews the direction of movement away from the target. As the task progresses, movements become more accurate and directional errors become smaller (Fig. 1C). Finally, the perturbation is often removed in a de-adaptation phase. Here, the new visuomotor mapping encoded during adaptation causes subjects to make errors in the opposite direction. The de-adaptation error is quickly corrected as subjects return to their original visuomotor patterns. Notably, the de-adaptation phase is sometimes referred to as the extinction phase or “wash-out” and reflects a recalibration to the original sensorimotor transformation rules as the new learned visuomotor routine is faded from memory.

## The Link Between Sensory Prediction Errors and the Cerebellum

The onset of the perturbation in visuomotor adaptation experiments can be sudden, which is likely to create explicit awareness. When the perturbation is gradually increased over time, processing is more implicit [9]. Similarly, research has shown that providing online visual feedback (i.e. showing the cursor during the movement) in a visuomotor adaptation to rotation task promotes more implicit processing when compared to end-point feedback [10, 11]. The seminal work by Mazzoni and Krakauer [12] suggested though, that multiple learning processes contribute to visuomotor adaptation.

Evidence has accumulated to support two systems that operate simultaneously in visuomotor adaptation: one is explicit, relying on cognitive strategies such as target aiming [11] and the other is implicit and dependent on the difference between a predicted sensory consequence and an actual sensory feedback, so called sensory prediction errors (SPE) [13]. SPE are used to continuously update an internal forward model to enable more accurate predictions for future movements [8]. Theoretical, neurophysiological, and behavioral studies support the notion that the cerebellum uses SPE to acquire and then fine-tune internal forward models of action [14–16]. The importance of the cerebellum to SPE-based visuomotor adaptation has been thoroughly investigated in patients with ataxia or cerebellar degeneration, thus contributing to our understanding of the underlying processes governing visuomotor adaptation.

One important study that established a link between the hypothesized cerebellar SPE processing and visuomotor adaptation asked whether the rate of adaptation is influenced by the addition of motor feedback through correctional hand movements compared to only SPE [17]. The authors found that adaptation to a visuomotor perturbation in both cerebellar ataxia patients and controls was not affected by online corrections, suggesting that visuomotor adaptation is driven by cerebellar-dependent SPE rather than by the motor correction. When provided with a cognitive strategy to compensate for the visuomotor rotation [12], by explicitly aiming for the neighboring target, healthy subjects showed continued SPE-based learning on top of the cognitively applied strategy. This led to a failure to reach the target. In contrast, patients with cerebellar degeneration were able to apply the strategy without interference by SPE [18]. These results further strengthen the link between SPE and the cerebellum.

Conflicting evidence arises when abrupt adaptation (leading to explicit awareness) is compared to a gradual exposure to a perturbation (implicit processing). Criscimagna-Hemminger and colleagues [19] observed similar deficits in cerebellar ataxia patients under abrupt adaptation, but when a gradual exposure to the force field was applied, patients were able to improve performance. Similarly, gradual adaptation to a visuomotor rotation was observed by Henriques and colleagues [20] in patients with lesions in the cerebellum due to stroke. Thus, these results suggest that implicit processing resulting from a gradual exposure to a perturbation may rely on structures outside of the cerebellum, such as the parietal cortex [21–23]. Schlerf and colleagues [24] observed, under both abrupt and gradual visuomotor rotation, marked deficits in patients with cerebellar ataxia compared to healthy controls. In accordance with this observation, Butcher and colleagues [25] showed that cerebellar ataxia patients were impaired not only in SPE-based visuomotor adaptation, both abrupt and gradual, but also had difficulties in forming explicit aiming strategies to counter the observed target error. Butcher et al. [25] interpreted their findings to indicate that the cerebellar deficit in adaptation is not purely a cerebellar phenomenon but also reflects dysfunction in cortico-cerebellar networks that play a role in cognitive processing. We will expand on this notion in the next section.

A recent study found, however, that when cerebellar ataxia patients see their hand during the task, which means that there is no SPE, they are able to develop and apply aiming strategies and improve their performance [26]. One way to understand this, as the authors explain, would be that in a typical visuomotor adaptation task such as in Butcher et al. [25], when the hand is not visible to the patient, there is a preference towards SPE-based learning over the more explicit target-error learning. That could explain why patients could not form explicit strategies even when instructed to do so. It is important to note that these findings are from older patients and may relate to their cognitive impairments [27]. To sum, different types of learning act in concert in visuomotor adaptation. Studies in cerebellar patients suggest that while the cerebellum plays an important role in the process of visuomotor adaptation, involvement of cortical structures such as the parietal cortex and the premotor cortex, could be as crucial and demand further investigation.

## Contribution of Cerebellar Sub-structures to Visuomotor Adaptation

Despite the important, yet conflicting, insights provided by these studies in cerebellar ataxia and cerebellar stroke patients, the heterogeneity of the affected sub-cerebellar structures and connected brain regions may obscure the actual role the cerebellum plays in this task. One strategy is to use imaging to explore the effect of cerebellar sub-structure pathology on deficits in visuomotor adaptation. For example, Rabe and colleagues [28] showed that adaptation to a rotation was associated with cerebellar regions that lie more posterior to the ones associated with force field adaptation. This result has been replicated and expanded to subjects with both chronic, acute and subacute lesions [29, 30]. These replications also helped to localize the areas related with different forms of adaptation more precisely: anterior cerebellar lobules IV and V were important for force field adaptation whereas lobule VI was more important for visuomotor rotation. This differentiation could be related to distinct connectivity patterns of anterior cerebellum with the primary motor cortex and posterior cerebellum with premotor areas and posterior parietal cortex [31, 32]. Thus, the anterior cerebellum receives somatosensory information that could be more critical for processes underlying force-field adaptation, whereas information transfer with the parietal cortex, which lies on the dorsal visual stream [33], could play an important role in visuomotor adaptation.

This body of evidence could thus explain some of the contradictory results obtained in pure behavioral studies in cerebellar patients (detailed above) and stress the contribution of posterior cerebellum and its interactions with premotor and parietal cortex to the process of adaptation to a visual rotation.

## Dynamic Changes in Cerebellar Activity in the Process of Visuomotor Adaptation

While structural imaging studies of the cerebellum show a dissociative role of anterior and posterior regions of the cerebellum on the specific visuomotor adaptation task, they can provide little insight into dynamic cerebellar involvement in different phases of visuomotor adaptation. Functional imaging studies in healthy subjects have provided evidence that activity in posterior cerebellar lobules is specifically associated with neural processes underlying early adaptation to a visuomotor rotation, while more anterior regions of the cerebellum could be responsible for maintaining long-term representations of the newly acquired visuomotor routines. For example, in an elegant study by Kim and colleagues [22], a computational model of visuomotor adaptation, accounting for multiple memories on different time scales, revealed that bilateral cerebellar crus I was associated with memories on a fast time-scale (a few seconds), while cerebellar lobule VI was associated with memories on a slow time-scale (a few hours). Most recently, Tzvi et al. [34] examined the dynamics of cerebellar activity during the early and late phases of visuomotor adaptation. The authors found that activity in left cerebellar crus II and lobule VI gradually decreased during adaptation to a visual rotation, and then rebounded when adaptation was suddenly removed during a de-adaptation phase. Thus, these studies demonstrate dynamic shifts in cerebellar activity in the process of visuomotor adaptation.

Dynamic involvement of cerebellum in visuomotor adaptation has been also investigated in animals using electrophysiological recording in the monkey’s cerebellum. These studies asked whether simple-spike activity of Purkinje cells (PC) encodes movement dynamics such as muscle activity, or movement kinematics such as velocity, direction and position of the movement, under adaptation to an external force-field. While Pasalar et al. [35] found no evidence that an external force field affects PC activity in lobules IV-VI, Yamamoto et al. [36] showed that PC activity in lobules V-VI differed depending on the type of force-field applied, suggesting that PC in lobules V-VI encode movement dynamics. Thus, it remains to be established whether PC simple-spike activity underlies movement dynamic or movement kinematics in visuomotor adaptation.

## Contribution of Cortico-striato-cerebellar Networks to Visuomotor Adaptation

Importantly, the cerebellum does not operate in isolation in the process of visuomotor adaptation. Imaging studies have demonstrated consistent activation of cortical structures such as motor, premotor, and parietal cortices as well as striatum in addition to cerebellum during visuomotor adaptation [21, 37, 38], indicating that these structures probably also play important roles in visuomotor adaptation. Together with the cerebellum, the parietal cortex has been suggested as well to play an important role in SPE, particularly when predicting the goals and plans of future movements [39]. For instance, Ferrari-Toniolo and colleagues [40] showed that neuronal activity in inferior parietal lobule of the primate encodes the direction of hand force changes under isometric conditions. Similarly, in humans, the superior parietal cortex encoded specifically the hand direction when subjects made pointing movements [41]. Parietal cortex, like other cortical areas, projects to the cerebellum via the pontine nuclei. The cerebellum projects back via the Thalamus. Thus, it is likely that this connectivity [42] is crucial for SPE in visuomotor adaptation.

This has motivated theoretical models of network interactions underlying visuomotor adaptation [43, 44]. Most recently, Haar and Donchin [32] suggested a multilayer model for motor control in which parallel interactions between specific cortical areas and respective regions in basal-ganglia and cerebellum govern different aspects of proprioception and movement. The authors propose based on their recent findings [21, 45], that in the context of a visuomotor adaptation task, the premotor cortex encodes the intended cursor movement (where do we want the cursor to go) and posterior parietal cortex represents the target and cursor position on the screen (where did the cursor actually go). These cortical nodes communicate with specific areas of the cerebellum to assist predictive computation of this representation.

**Fig. 2:**
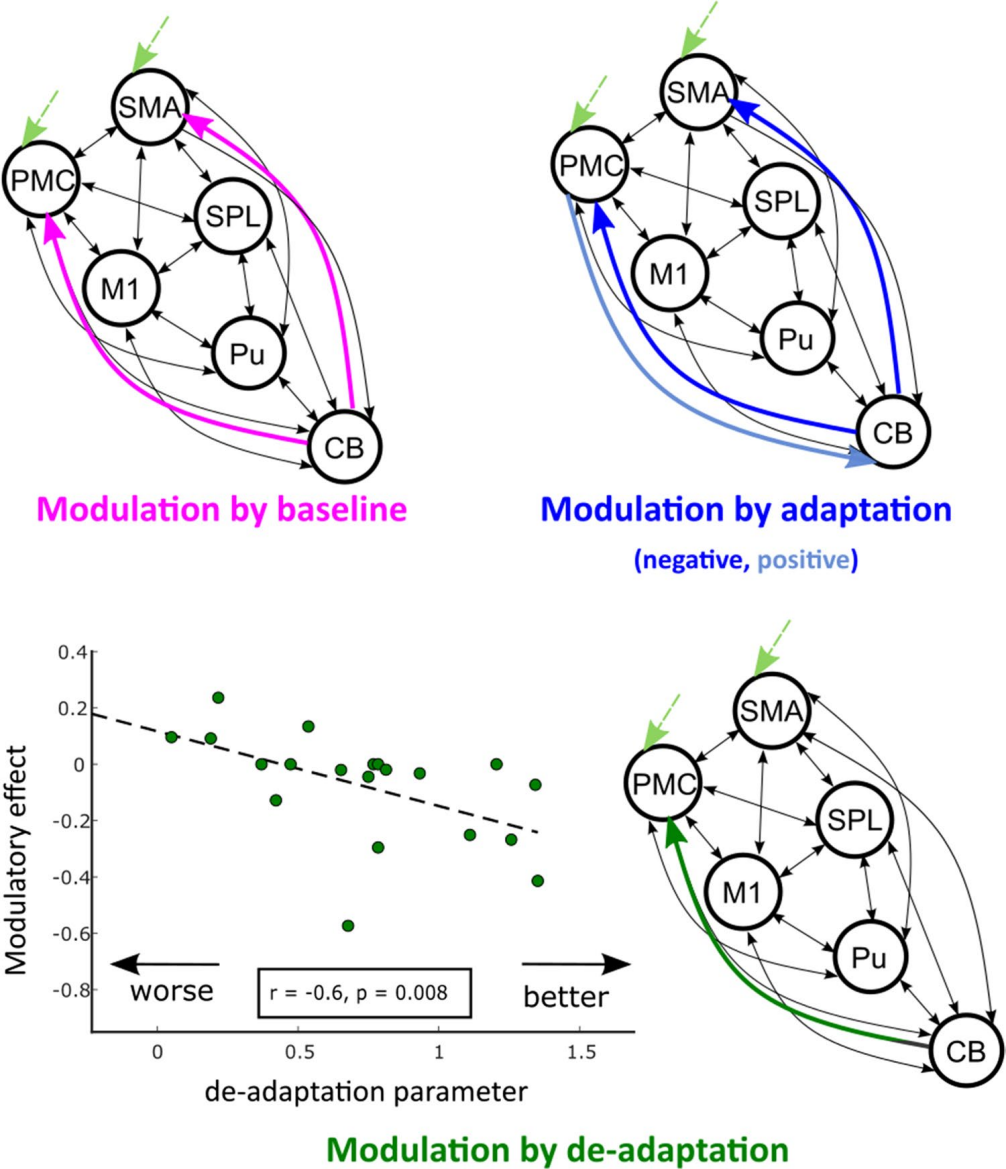
Network interactions underlying visuomotor adaptation (adapted from [34]). Shown is the cortico-striato-cerebellar network (left hemispheric, right cerebellum) modulated by visuomotor adaptation. CB: cerebellum, Pu: putamen, SPL: superior parietal lobule, M1: primary motor cortex, PMC – dorsolateral premotor cortex, SMA: supplementary motor area. In color are directed connections shown by dynamic causal modelling and Bayesian model selection procedure to be modulated by the different task conditions (baseline, adaptation and de-adaptation). Note that CB—> PMC is always negatively modulated by the different conditions, while PMC—> CB is positively modulated by adaptation only. Modulation of CB – > PMC connection by de-adaptation was more negative (left-bottom panel) the faster subjects returned to the original visuomotor routine

In the study by Tzvi et al. [34] discussed above, the authors used dynamic causal modeling to test the above proposed interactions between cortical structures and cerebellum during visuomotor adaptation. The authors examined interactions within a cortico-striato-cerebellar network by comparing 21 models with different connectivity patterns between M1, dorsolateral premotor cortex, supplementary motor area, superior parietal lobe, putamen and cerebellum. The results show consistent negative modulation of interactions from cerebellum to dorsolateral premotor cortex and supplementary motor area during both baseline and adaptation blocks (Fig. 2). That is, the connection from cerebellum to dorsolateral premotor cortex remained consistent whether the cursor movement was rotated or not. De-adaptation was associated with negative modulation of cerebellar to dorsolateral premotor cortex connectivity. In fact, the degree of negative modulation of this connection predicted the rate of behavioral de-adaptation (Fig. 2). Notably, a positive modulation of connection from dorsolateral premotor cortex to cerebellum was unique for adaptation. Under the theory propounded by Haar and Donchin [32], the observed cerebellar-dorsolateral premotor cortex connectivity changes can be interpreted as expression of the updating of an internal model representing the transformations of hand movements to cursor movements in the context of task demands.

Another possible explanation of these results supports the idea that visuomotor adaptation integrates multiple learning systems [12]. It is possible that an interplay between dorsolateral premotor cortex, representing the explicit, strategic component of visuomotor adaptation, and cerebellum, representing the implicit, SPE-based learning component, is at the base of visuomotor adaptation, leading to formation of a new internal forward model in the cerebellum. Future studies could attempt to disentangle processes underlying visuomotor adaptation using experimental designs such as in the work by Mazzoni and Krakauer [12] and investigate the underlying interactions between the cerebellum and dorsolateral premotor cortex, as well as other frontal regions, suggested to be key for the strategic learning component.

## The Effect of Non-invasive Stimulation of the Cerebellum on Visuomotor Adaptation

To go beyond purely correlational relationships between cerebellar function and visuomotor adaptation, studies have employed non-invasive stimulation techniques. These include among others, transcranial direct current stimulation (tDCS), a method thought to modulate synaptic plasticity in a target region. For example, Galea et al. [46] showed that anodal tDCS over the right cerebellum led to faster adaptation to a visuomotor rotation as evident by rapid reduction of movement errors compared to sham or to anodal tDCS to left M1. Later studies have replicated this finding with the same task [47, 48], in older subjects [49], using force field adaptation [50] as well as in other forms of motor skill learning that require adaptation to a visuomotor manipulation [50–53]. However, recent attempts to replicate the positive effect of cerebellar tDCS on visuomotor adaptation in large groups of subjects were not successful [54–56]. Overall, the results cast doubt on the use of cerebellar tDCS as a practical tool to affect visuomotor adaptation. Authors of the papers reporting no effect of cerebellar tDCS remarked on the high inter-individual variability. The large variability might have different causes: it might be driven by individual reactions to tDCS [57] or it might be driven by individual ability to react to the visuomotor manipulation. Possibly, inter-individual differences may also arise from variability in connectivity patterns between the cerebellum and premotor cortex as well as parietal cortex that may influence visuomotor adaptation ability in individual subjects. Thus, future studies could attempt to reconcile these results by individualizing the tDCS protocol based on underlying neuroanatomy using computational modelling [58].

Other forms of cerebellar non-invasive stimulation may also hold potential in impacting cerebellar activity during visuomotor adaptation. A recent study employed theta burst stimulation, a form of repetitive transcranial magnetic stimulation, to the posterior cerebellum shortly before a visuomotor adaptation task. Results showed that an intermittent protocol (iTBS) led to speeding up of error reduction in adaptation to a visual perturbation [59], whereas a continuous protocol (cTBS) led to the opposite effect, suggesting that cerebellar TBS could also be useful as a tool to focally modulate cerebellar function during visuomotor adaptation. Here as well, careful consideration of inter-individual differences in connectivity patterns between the cerebellum and premotor as well as parietal cortex should be accounted for. Diekhoff-Krebs and colleagues showed that stroke patients who received iTBS to M1 had better motor performance depending on connectivity between M1 and SMA prior to the intervention [60]. Thus, it is likely that depending on the task at hand, connectivity patterns could influence the effect of transcranial magnetic stimulation on behavioral performance.

To sum up, a potential for non-invasive stimulation to drive a causal understanding of visuomotor adaptation remains, but success may depend on application of the right non-invasive stimulation protocol. One promising direction is the combination of non-invasive recording such as fMRI and non-invasive stimulation [61] which could enable assessment of the stimulation effect depending on inter-individual differences in connectivity patterns.

## Concluding Remarks

In this mini-review, we summarized current evidence linking the cerebellum to neural processes underlying visuomotor adaptation. Importantly, we demonstrated that the cerebellum does not act alone, but probably serves as an important hub in a network comprising of the premotor cortex and the parietal cortex that acts coherently to adjust motor routines in response to dynamic changes in the environment.
